# Defining Smallness for Gestational Age in the Early Years of the Danish Medical Birth Registry

**DOI:** 10.1371/journal.pone.0016668

**Published:** 2011-01-31

**Authors:** Rasmus á. Rogvi, Rene Mathiasen, Gorm Greisen

**Affiliations:** Department of Neonatology, Copenhagen University Hospital, Rigshospitalet, Copenhagen, Denmark; University of Giessen Lung Center, Germany

## Abstract

**Background:**

Being born small for gestational age (SGA) is associated with decreased insulin sensitivity and increased blood pressure in childhood, but the association with clinical disease in early adulthood is less certain. The Danish Medical Birth Registry has registered all births in Denmark since 1973, but due to variable data quality, data is most often used only from 1981 onwards, and birth registers in other countries may have similar problems for the early years. We wanted to examine whether the data can be used for identification of children born SGA and used in future research.

**Methodology/Principal Findings:**

All persons born between 1974 and 1996 were identified in the Danish Medical Birth Registry (n = 1.704.890). Immigrants and children without data on gestational age and birth weight were excluded, and a total of 1.348.106 children were included in the analysis. The difference between the different variables used in the history of the registry were examined, and the quality of data in the birth registry from 1974-1981 was examined and compared to subsequent years.

Data on birth weight and gestational age in the early years of the registry is inconsistent, and the identification of children born SGA is inaccurate, with 49% false-positives. The biggest source of error is due to the rough and inaccurate intervals used for gestational age. By using –3 standard deviations as a cut-off for the identification of children born SGA, the number of false-positives was reduced to 9%, while the amount of false-negatives were increased.

**Conclusion:**

Choosing –3 standard deviations for identifying children born SGA is a viable, though not optimal solution for identifying children born SGA. Overall the data in the registry is of sufficient quality to be used in further medical research.

## Introduction

Children born small for gestational age (SGA) have previously been reported to have an increased risk of infant mortality, cerebral palsy, cardiovascular disease, insulin dependent diabetes mellitus (IDDM), non insulin dependent diabetes mellitus (NIDDM) and metabolic syndrome [1]–[11]. The association between being born SGA and the risk of diseases in adulthood is still unclear. In recent years several studies have been published discussing metabolic programming in fetal life, and the subsequent risk of morbidity in adulthood. Of special interest are the studies from the Dutch Famine Birth Cohort Study, which examines the effects of children exposed to malnutrition in utero. These children have been shown to have a decreased insulin secretion and a more atherogenic lipid profile, though the risk of developing metabolic syndrome was not shown to be increased [12]–[14].

The Danish Medical Birth Registry offers a tool for epidemiological research on the population level. The Danish Medical Birth Registry has registered all births in Denmark since 1973, holding data on birthweight and gestational age among others. The data in the early years from 1973 to 1981, however, are imperfect, coding birthweight and gestational age into intervals, probably due to data storage being expensive at the time. Furthermore some birth cohorts have a substantial amount of missing data [15]. Birth registers in other countries may suffer from similar limitations.

Several studies using The Danish Medical Birth Registry have excluded the early birth cohorts, some citing the inconsistencies as a reason [3], [16].

The early birth cohorts are of interest first of all because they approach an age where the incidence of NIDDM and cardiovascular disease increases markedly and secondly because these birth cohorts are the largest in recent times in Denmark, including over 500.000 births from 1973 to 1981. Thus we examined whether they can be compared to subsequent birth cohorts and included in epidemiological research with a focus on smallness for gestational age. This is a very active research field and other early birth register cohorts may have similar limitations.

## Materials and Methods

Data were extracted in December 2006 from the Fertility Database held by The National Board of Health in Denmark. The registers include data on birthweight, gestational age, place of birth, and Apgar score. Data was extracted for a yet unpublished study on late effects of brain injury in neonates, and this is an opportunistic study using the same data.

All children born in Denmark as well as legal immigrants are assigned a civil personal registration number (CPR number). The CPR number is a 10-digit number, with the 6 first digits being the date of birth, and the last four being a unique identifier for each person. The number is used for personal identification and registration in many public and private instances, and allows linking data from different registries in medical research.

The data collection for the study was approved by the Danish Data Protection Agency. The CPR-numbers were encrypted and thus anonymised for the researchers. Under Danish legislation it is not necessary to apply for approval by The Danish National Committee on Biomedical Research Ethics for database studies as long as the study is approved by the Danish Data Protection Agency. Neither is it necessary to get written consent from individuals for database studies.

We identified 1.704.890 people in the Danish CPR register born between 1974 and 1996. 1.545.641 persons were registered in The Danish Medical Birth Registry, the remainder primarily being immigrants (n = 159.249).

We obtained the recorded birthweight and gestational age and individuals without data on both were excluded (n = 189.586, 12.2%) as well as stillbirths (n = 7.206, 0.47%). To exclude unlikely values, birthweight by gestational age (GA) was compared to the expected value as calculated by the use of Marsal's charts of normal intrauterine growth [17]. Children with a birthweight ± 5 SD of the expected value were excluded (n = 743, 0.06%). Data on a total of 1.348.106 individuals were analysed.

### Variables

#### Birthweight

From 1974 to 1996 birthweight was coded in the variable *vegt1* in 250-gram intervals (501–750 grams, 751–1000 grams etc.). In our analysis we assigned the centre value of the interval as the specific birthweight for all individuals.

From 1979 onwards, the variable *vaegt* was the primary birthweight variable. From 1979 to 1991 *vaegt* was coded in 10-gram intervals and afterwards in 1-gram intervals.

#### Gestational Age

From 1974 to 1977 gestational age was coded in the variable *fuldb*, which corresponds to term/preterm birth. The value 0 is term birth (according to the current documentary of the registry this means gestational age ≥ 40 weeks), and the values 1–8 define increasing degrees of prematurity (table 1) [15].

**Table 1:** Expected and observed number of children in each gestational age interval group, children born 1974–1977.

Gestational age group	Corresponds	Nominal GA	Observed % of newborn	Expected % of newborn^a^
0	Term birth	≥ 40	89.6	70.0
1	1 week preterm	39	1.1	13.2
2	2 weeks preterm	38	2.9	7.8
3	3 weeks preterm	37	2.4	3.8
4	4 weeks preterm	36	1.5	2.1
5	5–6 weeks preterm	34–35	1.0	1.6
6	7–9 weeks preterm	31–33	0.6	1.0
7	10–12 weeks preterm	28–30	0.3	0.4
8	More than 12 weeks preterm	<28	0.2	0.21

a *Compared to observed values in 1978.*

From 1978 onwards, the variable *svlengde* was used, corresponding to the gestational age in completed weeks.

### Analysis

Data was analysed with SAS 9.1.3 (SAS Institute, Inc, Cary, NC). The mean values and the difference between the two weight variables were calculated for each year and gestational week.

A graphical representation of the 1-gram weight variable was made with complete histograms for each year, to assess whether there were preferences for certain values.

When GA given in completed weeks was converted to GA in days, we multiplied by 7 and added 3 to approximate the mean number of gestational days for the week interval.

Expected birthweight was calculated using the sex-specific models published by Marsal [17]. Children <-2 SD of expected birthweight were classified as SGA, as used in the article by Marsal. Using PROC GLM in SAS we constructed a 4^th^ power model of birthweight per gestational age (to mimic Marsal's 4^th^ power model) for the years 1974 to 1977 to estimate a more correct distribution of gestational age for the different values.

The trend in weight per GA was graphed for the two GA variables. For each gestational week, development in birthweight was modelled with linear regression using the PROC GLM procedure.

For the children born in 1979 we pooled the children born at GA 38–45 in one single group to examine the effect of the GA classification used from 1973 to 1977. We identified all children with a birthweight less than –2 and –3 SD of expected in both groups. Afterwards we compared the classification of children born as SGA in the pooled group with their classification using their true GA in completed weeks.

## Results

### Birthweight

**Figure 1:**
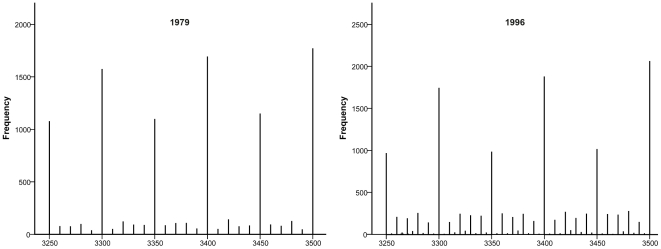
Histograms of birthweight in 1979 and 1996. Values divisible by 50 and 100 continued to dominate in spite of coding of birthweight was done in 1-gram intervals since 1992.

In 1979 the birthweight was coded in 10-gram intervals, but there was a distinct preference for 100- and 50-gram values (figure 1). In 1996 where birthweight was coded in 1-gram intervals this preference persisted, though more 10-gram values were registered.

The mean value of the 1-gram variable was higher than the 250-gram variable for all years, though the difference decreased from approximately 22 grams in 1979 to 16 grams in 1996.

For intervals below the mean, the 1-gram value had a higher mean interval value than the 250-gram value, while the opposite was true for intervals above the mean (data not shown).

### Gestational age

From 1974 to 1977 the gestational age was coded in the birth register as seen in table 1. The number of births coded with GA value  = 0, i.e. 40 weeks and above, was nearly 90%, which exceed the percentage coded as 40 weeks and above in the following years by almost 20%.

**Figure 2:**
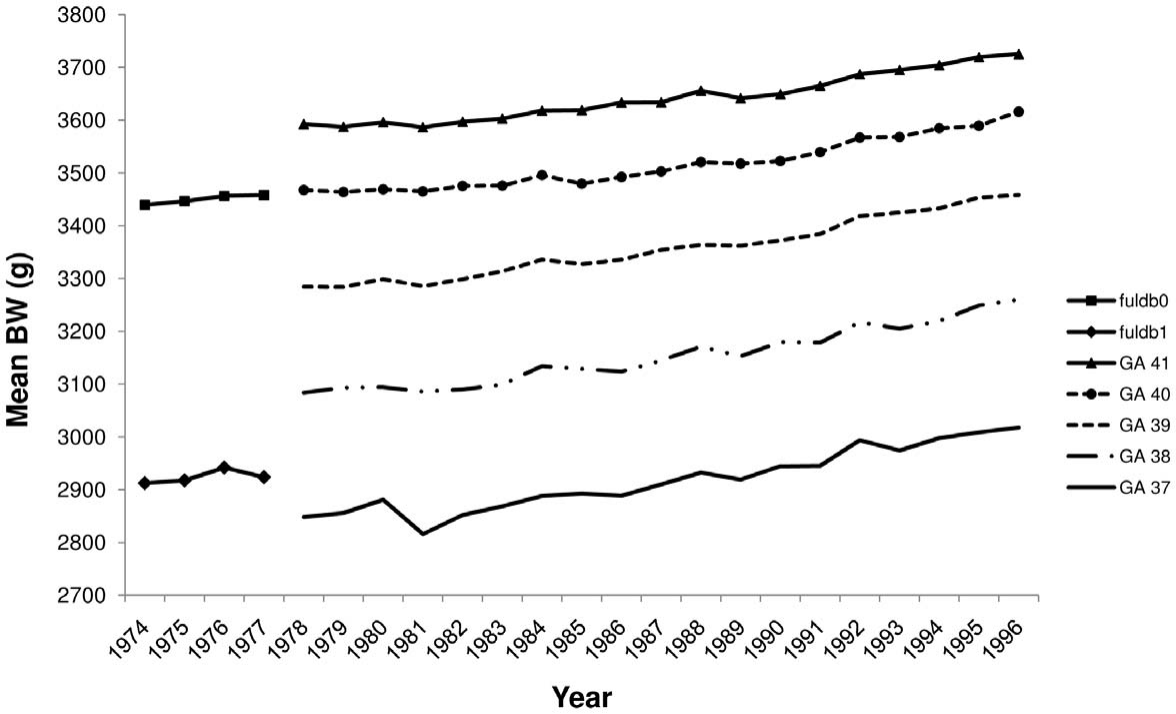
Mean BW per year. Gestational age specific birthweight in the period 1974 to 1996. A gradual increase is seen. Gestational age was coded in the variable *fuldb* during the years 1974 to 1977. *fuldb0* meant ≥40 weeks and *fuldb1* meant 39 weeks. In reality, it appears that the mean gestational age of the children coded 35 years ago as *fuldb0* was almost exactly 40 weeks and the children coded as *fuldb1* was between 37 and 38 weeks.

When correcting for gestational age in studies it is necessary to recode these values to a gestational age in days or weeks. When comparing the weight for the GA value  = 0 age to GA in weeks, it follows the trend in weight for week 40 (figure 2). When doing the same for the value 1, the curve does not fit with week 39 as expected. If comparing the value to GA 37 the mean birthweight appears to be 75 grams (2.6%) too high, whereas comparing to GA 38 it appears to be 160 grams (5.5%) too low.

We made a model of birthweight per GA in 1978 and calculated the corresponding week in the regression model when using the 1977 mean birthweight (table 2).

**Table 2:** Mean birthweight of children born 1974–1977 in each gestational age interval group.

Gestational age group	Nominal GA	Mean birthweight ± SD	Calculated GA^a^
0	≥40	3458±473	40.0
1	39	2924±454	37.4
2	38	2813±420	36.9
3	37	2679±441	36.4
4	36	2448±402	35.4
5	34–35	2155±383	34.2
6	31–33	1770±368	32.5
7	28–30	1330±318	29.8
8	<28	979±251	26.3

a *Given the distribution of birthweight in 1978.*

In 1978 the procedure of reporting gestational age to the Danish Medical Birth Register changed. This resulted in a significant increase in the number of cases without data on GA. There are only small differences in the mean birthweight between cases with and those without data on GA (table 3). The distribution of GA or mean birthweight did not differ systematically between the two groups from 1978 to 1981. From 1982 onwards the number of cases with data missing on GA was less than 1%.

**Table 3:** Birthweight on children with and without data on gestational age.

Year	Missing data on GA	Mean birthweight on children with GA data	Mean birthweight on children without GA data
1978	30.5%	3351±586	3385±566
1979	26.5%	3376±573	3384±604
1980	20.3%	3373±577	3375±644
1981	13.2%	3369±599	3405±554

The mean gestational age was reduced by approximately 2 days from 1978 to 1996, and using Pearson's test for trend for GA 34–39 we found a significant positive correlation between year and birth week frequency (p<0.0001 for all weeks except 36 where p = 0.021), whereas there was a strong negative correlation (p<0.0001) between year and frequency for GA 40.

### Weight for gestational age

The percentage of children with a birthweight less than -2 SD of expected were calculated using the gestational ages for the nominal and the calculated model shown in table 2. In 1974–77 the percentage of SGA children were distributed as seen in table 4. After 1979, using the 1-gram variable instead of the 250-gram variable yielded a decrease in the total number of children regarded as SGA from 5.0% to 4.3% (p<0.0001).

**Table 4:** Percentage of children born SGA.

Gestational age group	Nominal model	Calculated model
0	5.6%	5.6%
1	21.3%	8.0%
2	21.7%	7.2%
3	14.3%	14.3%
4	30.0%	13.2%
5	38.4%	38.4%
6	26.0%	54.8%
7	41.0%	41.0%
8	41.0%	16.8%

**Figure 3:**
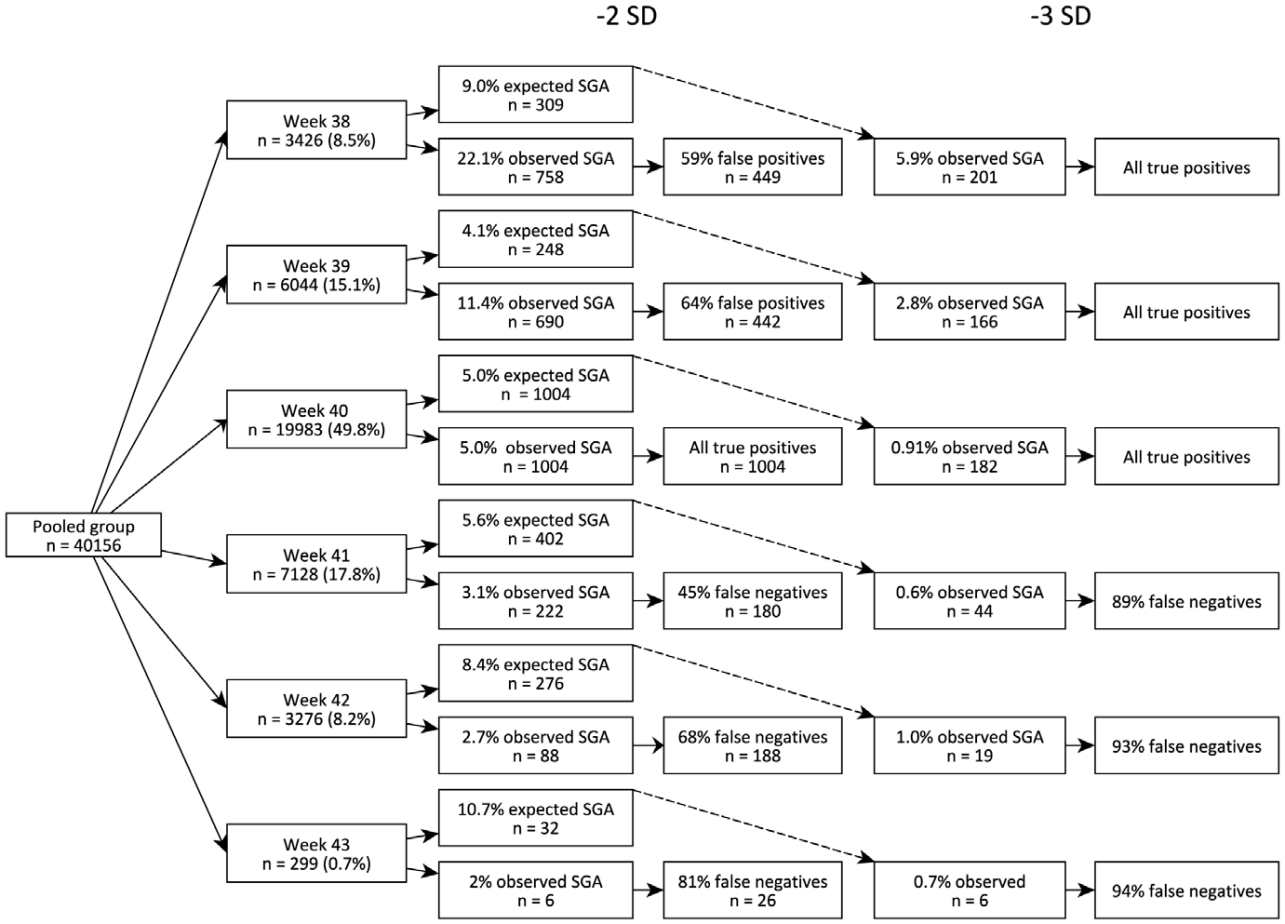
Estimation of SGA misclassification of children born in 1974–77. The births in 1979 with gestational age 38–45 were classified as SGA using - 2SD and - 3SD thresholds. The week specific thresholds (expected SGA) as well as common 40 week thresholds (observed SGA) were used. Use of the - 2SD threshold yielded both false positives and false negatives. Use of the - 3SD threshold greatly reduced the number of false positives. Week 44 and 45 are not shown due to the small amount of children born these weeks.

The distribution in table 1 suggests that the GA  = 0 value covers not only children born between GA 40 and 45, but also a lot of the children born week 38 and 39. For the 1979 birth cohort we pooled children born in GA 38–45 in one single group and compared the number of children regarded as SGA if they were assumed to be born week 40 and classified using the 250-gram interval, to the number of children assumed to be SGA considering their gestational age in completed weeks classified using the 1-gram interval (figure 3).

The total number of children classified as SGA, using the standard –2 SD as a cut-off, increased from 4.8% to 6.9% when the children were pooled. In the pooled group 49% of the SGA population were false positive SGA. When using –3 SD as a cut-off, the number of false positives was reduced from 49% to 9%. Those still false-positive had a mean SD of -1.8. The number of false-negatives was increased to 62% of the SGA population, and the total number of children classified as SGA was reduced from 6.9% to 2%. The mean SD was reduced with 0.25 SD from -2.47 SD to -2.72 SD.

## Discussion

Our results show that data from the early years of the Danish Medical Birth Registry is of mediocre quality but can be used in further research with care.

The problems are:

- A preference for 100- and 50-gram values when reporting birthweight
- A classification of gestational age in intervals
- Years with many children missing data on gestational age
- A small difference in mean birthweight in children with and without data on gestational age
- Due to the first and second problem there are difficulties in classifying children as SGA

These finding are in accord with other studies using data from these years, though the approach to overcome them have differed [3], [16], [18].

### Problems with coding of birthweight

The preference seen for 100- and 50-gram values in the early years could be due to the use of mechanical scales, and would thus be expected to disappear as digital scales were more frequently used. As the preference persisted in 1996 where most Danish hospitals had switched to digital scales, it seems to be due to rounding when reporting the weight, rather than limitations in the scales.

The old 250-gram variable tended to underestimate the weight of children with a birthweight lower than the mean, as expected given the approximately normal distribution of birthweight. The underestimation was reduced from 22 grams in 1979 to 16 grams in 1996. Overall, using the 250-gram variable instead of the 1-gram variable increased the number of children regarded as SGA by about 15%. The difference is small, but must be taken into consideration if comparing data directly between the two birthweight variables.

### Problems with coding of gestational age

From 1978 to 1981 data on gestational age is missing on 13–30% of children. We found a small difference in birthweight between the groups, with a tendency for the children with missing data on gestational age being slightly heavier, though the distribution of birthweight or gestational age did not differ systematically between the two groups. Therefore it appears safe to consider the births with data on gestational age an unbiased sample of those birth years. The coding of gestational age in the years 1974 to 1977 was very insufficient. Not only was term birth coded in a very wide gestational age interval, coding practice was also inconsistent with the documentation of the Danish Birth Register. For preterm children the gestational age coding is more accurate.

### Problems with classification as SGA

We chose to use a normal fetal growth standard with a -2 SD cut-off, since this is more likely to represent optimal/suboptimal fetal growth, instead of a cut-off classifying children below the 10^th^ percentile of the birth weight distribution as SGA. Comparing birthweight from 1974 to 1996 to Marsal's curves of birthweight, which are based on children born 1986–87, however, may not be optimal since the mean birthweight changes over time. From 1974 to 1986 the mean birthweight for GA 40 and 37 increased by 53 and 122 grams, respectively, corresponding to an underestimation of weight per gestational age of –0,12 and –0,25 SD if using the 1986 birth curves on the 1974 cohort. On the other hand, considering optimal growth as an absolute standard, at least some of this difference may be considered real.

The classification of SGA children was more severely obstructed by the coding of both birthweight and gestational age in wide intervals. This resulted in almost 50% of the population nominally SGA, being false positive. Using a threshold of –3 SD, however, identified 5000 individuals as being SGA of a cohort of over 270.000 Danes, aged 34–37 years at present. Only 9% of these 5000 individuals were false-positive SGA, with a mean SD of -1.8, and the rest had a birthweight that was truly less than – 2 SD for their gestational age. This SGA group, however, had a mean GA that is lower than expected and this bias may be a problem in contexts where gestational age is important.

The mean SD in this group was 0.25 SD lower than expected, which could signify that the identified cohort is more severely growth restricted than expected using -2 SD as a cut-off.

Danish national registries hold much medical, educational and social information, allowing large-scale epidemiological studies by linking the CPR numbers. A study using SGA as defined above in the birth cohorts from 1974 to 1977, on a condition with a diagnosed prevalence of 1%, would have a 91.9% probability of detecting a relative risk of 1.5 (confidence interval 95%) between this SGA group and the general population.

The overrepresentation of individuals born between 37 and 40 weeks in the SGA-group will constitute a significant bias for the study of outcomes such a neonatal respiratory morbidity, other neonatal complications, as well as short- and long term neuropsychological outcomes, for all of which even modest prematurity is an important risk factor [19]–[22]. For the study of metabolic illness the bias is likely to be less pronounced [22], [23].

In conclusion, it is possible to identify a large cohort of young adults born SGA in the years 1974–1977. The cohort exhibits GA bias and has a mean SD that is -0.25 SD lower than expected. Given the expected bias, this cohort can not be used as a replacement for later and more precise birth cohorts, but may be a valuable supplement.
